# Identification and Structural Characterization of a New Three-Finger Toxin Hemachatoxin from *Hemachatus haemachatus* Venom

**DOI:** 10.1371/journal.pone.0048112

**Published:** 2012-10-29

**Authors:** Vallerinteavide Mavelli Girish, Sundramurthy Kumar, Lissa Joseph, Chacko Jobichen, R. Manjunatha Kini, J. Sivaraman

**Affiliations:** 1 Department of Biological Sciences, Faculty of Science, National University of Singapore, Singapore, Singapore; 2 Department of Biochemistry, Medical College of Virginia, Virginia Commonwealth University, Richmond, Virginia, United States of America; Universidad Nacional Autonoma de Mexico, Instituto de Biotecnologia, Mexico

## Abstract

Snake venoms are rich sources of biologically active proteins and polypeptides. Three-finger toxins are non-enzymatic proteins present in elapid (cobras, kraits, mambas and sea snakes) and colubrid venoms. These proteins contain four conserved disulfide bonds in the core to maintain the three-finger folds. Although all three-finger toxins have similar fold, their biological activities are different. A new three-finger toxin (hemachatoxin) was isolated from *Hemachatus haemachatus* (Ringhals cobra) venom. Its amino acid sequence was elucidated, and crystal structure was determined at 2.43 Å resolution. The overall fold is similar to other three-finger toxins. The structure and sequence analysis revealed that the fold is maintained by four highly conserved disulfide bonds. It exhibited highest similarity to particularly P-type cardiotoxins that are known to associate and perturb the membrane surface with their lipid binding sites. Also, the increased B value of hemachotoxin loop II suggests that loop II is flexible and may remain flexible until its interaction with membrane phospholipids. Based on the analysis, we predict hemachatoxin to be cardiotoxic/cytotoxic and our future experiments will be directed to characterize the activity of hemachatoxin.

## Introduction

Snake venoms are rich sources of biologically active proteins and polypeptides [1]. Apart from its crucial role in paralyzing and digesting prey, snake venom is also an excellent source for novel toxins. Understanding the mechanisms of action of unique toxins, helps in the discovery of novel receptors and in the development of lead therapeutic molecules [2], [3]. Snake venom toxins can be broadly categorized as enzymatic and non-enzymatic proteins. They are also classified into various toxin superfamilies. Each superfamily contains structurally similar toxins that exhibit varied pharmacological activities. Some of the well characterized superfamilies of snake venom proteins include three-finger toxins (3FTxs), C-type lectin like proteins (CLPs), phospholipase A_2_s (PLA_2_s), serine proteases and metalloproteases [4]–[6]. 3FTxs, non-enzymatic snake venom proteins, are the most abundant toxins found in elapid (cobras, kraits, mambas and sea snakes) and colubrid venoms [4], [7]. Besides they have been reported from viperid venoms [8], [9]. 3FTxs are composed of 60–74 amino acid residues and 4–5 disulfide bridges. Structurally, all 3FTxs have a stable fold with three β-stranded loops extending from a central core containing all four conserved disulphide bridges, resembling the three fingers of a hand, and hence their common name [10], [11]. The conserved cysteine residues, along with invariant residues, such as Tyr25 and Phe27, contribute to proper folding [12]. Some 3FTxs have an additional fifth disulfide in loop I and II as in the case of non-conventional toxins and long-chain neurotoxins, respectively [11], [13]. In general, 3FTxs exist as monomers. However, a few of them exist as homo- or hetero-dimers in which the subunits are held together by either non-covalent interactions or by covalent (disulfide) linkages. For example, κ-bungarotoxin [14] and haditoxin [15] exist as non-covalent homodimers where the individual subunits are structurally related to long-chain and short-chain neurotoxins, respectively. The individual subunits are arranged in anti-parallel orientation and are held together mostly by hydrogen bonds between main-chain and side-chain atoms [15]–[17]. On the other hand, covalently linked 3FTxs include the homodimeric α-cobratoxin (α-CT) [18] and the heterodimeric irditoxin [19]. The structural analysis of the homodimeric α-CT [18] reveals the presence of a β-strand swap as well as two disulfide linkages between loop I of the individual subunits, thereby stabilizing the entire dimeric structure [20]. In irditoxin, the individual subunits are covalently linked through a single disulfide bond between loop I (of irditoxin B) and loop II (of irditoxin A) [19]. 3FTxs also exhibit minor structural variations in the length and conformation of the loops, and presence of longer C-terminal or N-terminal extensions (for details, see [4]). Despite overall similar fold, 3FTxs recognize a broad range of distinct molecular targets resulting in diverse biological activities [21], [22]. Based on their biological properties, 3FTxs can be classified as postsynaptic neurotoxins targeting the nicotinic [23] and muscarinic [24] acetylcholine receptors, cardiotoxins/cytotoxins targeting phospholipid membranes [25], fasciculins targeting acetylcholinesterase (AChE) [26], calciseptins and FS2 toxins targeting L-type calcium channels [27], [28], anticoagulants like naniproin, exactin and siamextin [R. M. Kini and colleagues, unpublished data] targeting various coagulation complexes, β-blockers like β-cardiotoxin targeting β_1_- and β_2_- adrenergic receptors [29], dendroaspin targeting α_IIβ_β_3_ (glycoprotein IIB-IIIa) [30], cardiotoxin A5 targeting α_v_β_3_ integrins [31] and antagonists of α_1A_ [32] and α_2A_ [33]adrenergic receptors. The ability of 3FTxs to recognize various molecular targets signifies the need for understanding structure-function relationships of these toxins. The three-finger fold is also observed in various other proteins like xenoxins from *X. laevis* [34] and HEP21 from hen egg white [35], as well as mammalian Ly-6 alloantigens [36], urokinase-plasminogen activator receptor [37] and complement regulatory protein CD59 [38]. 3FTxs in snake venoms are thought to be evolved from non-toxic ancestral proteins through gene duplication and accelerated evolution [39], [40].

**Figure 1:**
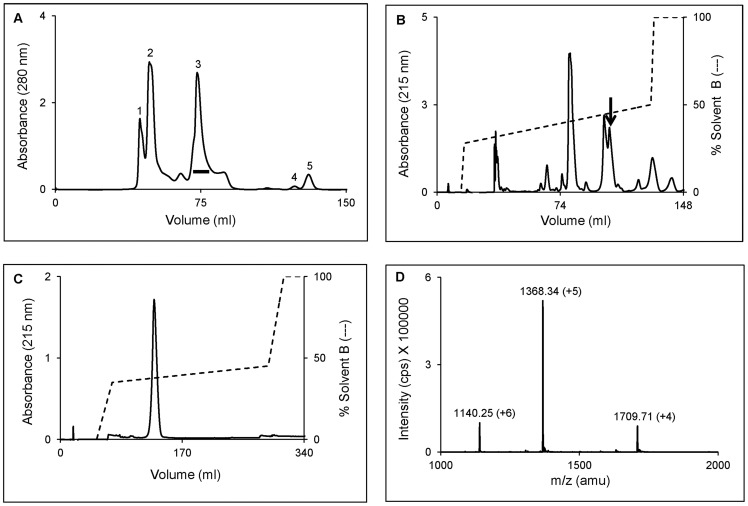
Purification of hemachatoxin from the venom of *H. haemachatus*. (**A**) Size-exclusion chromatogram of the crude venom. The proteins were eluted using 50 mM Tris-HCl, pH 7.4 and monitored at 280 nm. The fractions of peak 3 (*black horizontal bar*) were pooled and sub-fractionated on RP-HPLC. (**B**) RP-HPLC chromatogram of peak 3 using a linear gradient of 28–50% solvent B. The elution was monitored at 215 nm. The *black arrow* indicates the elution of hemachatoxin. (**C**) The re-purification of hemachatoxin on a shallow gradient of 35–45% solvent B. The elution was monitored at 215 nm. (**D**) The ESI-MS profile of hemachatoxin showing the three peaks of mass/charge (m/z) ratio ranging from +4 to +6 charges. The mass of hemachatoxin was determined to be 6835.68±0.94 Da.

In continuation of our efforts to understand the relationship between the structure and function of 3FTxs [4], [7], [15], we isolated, purified and determined the complete amino acid sequence and the crystal structure of a new three-finger toxin (hemachatoxin) from *H. haemachatus* (Ringhals cobra) venom at 2.43 Å resolution. The overall fold of hemachatoxin is similar to other known 3FTxs. The structure and sequence analysis revealed that the fold is maintained by four conserved disulfide bonds. Our efforts on the structure and sequence analyses combined with literature suggested that the unique biological activities of the 3FTxs are associated with the subtle conformational differences in the three β-strand loops. In addition, our analysis suggests that hemachatoxin might be endowed with cardiotoxic/cytotoxic activity.

**Figure 2:**
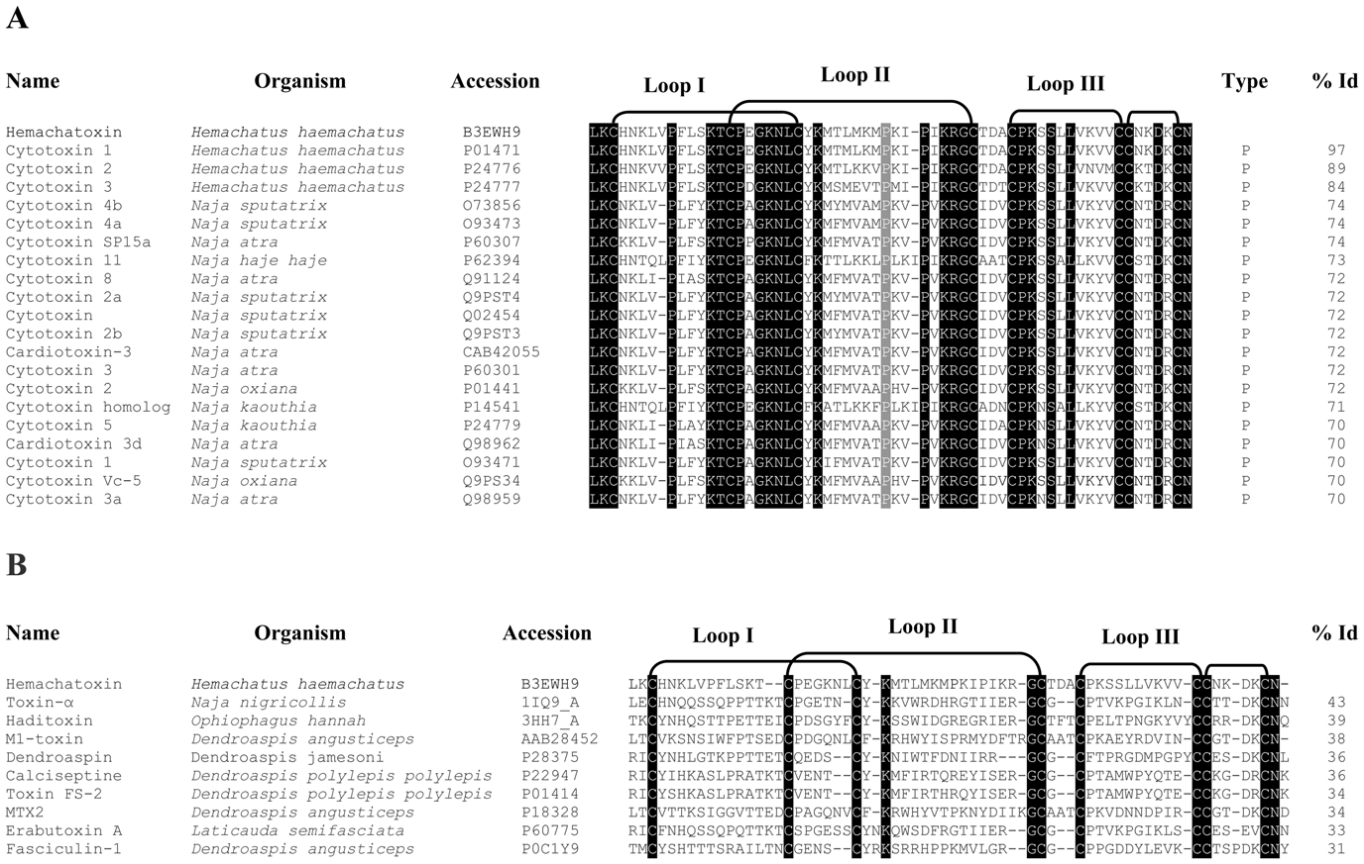
Multiple sequence alignment of hemachatoxin with cardiotoxins/cytotoxins (A) and other three-finger toxins (B). Toxin names, species and accession numbers are shown. Conserved residues in all the sequences are highlighted in black. The type of cardiotoxin based on the conserved Pro31 is highlighted in grey. Disulfide linkages and loop regions are also shown. The sequence identity (in percentage) of each protein with hemachatoxin is shown at the end of each sequence.

**Table 1:** Crystallographic data and refinement statistics.

Data collection*	
Unit Cell (Å)	a = 49.7, b = 50.1, c = 57.8
Resolution range (Å)	50-2.43 (2.47-2.43)
Wavelength (Å)	1.5418
Observed reflections	28936
Unique reflections	5614
Completeness (%)	96.2 (84.5)
Redundancy	3.9 (3.7)
aR_sym_	0.06 (0.17)
I/SigI	20.6 (11.7)
**Refinement**	
Resolution range (Å) I>σ(I)	30–2.43
bR_work_	0.23
cR_free_	0.28
Root mean square deviation	
Bond lengths (Å)	0.008
Bond angles (°)	1.377
Average B-factors (Å^2^)	
Protein atoms (938 atoms)	40.30
Water molecules (62 atoms)	37.1
Wilson B value	36.54
**Ramachandran statistics**	
Most favored regions (%)	98.31
Allowed regions (%)	1.69
Disallowed regions (%)	0

Statistics from the current model.

a R_sym_ = Σ|I_i_−<I>|/Σ|I_i_| where I_i_ is the intensity of the i^th^ measurement, and <I> is the mean intensity for that reflection.

b R_work_ = Σ| F_obs_−F_calc_|/Σ|F_obs_| where F_calc_ and F_obs_ are the calculated and observed structure factor amplitudes, respectively.

c R_free_ = as for R_work_, but for 10.0% of the total reflections chosen at random and omitted from refinement.

* Values in the parenthesis are the highest resolution bin values.

**Figure 3:**
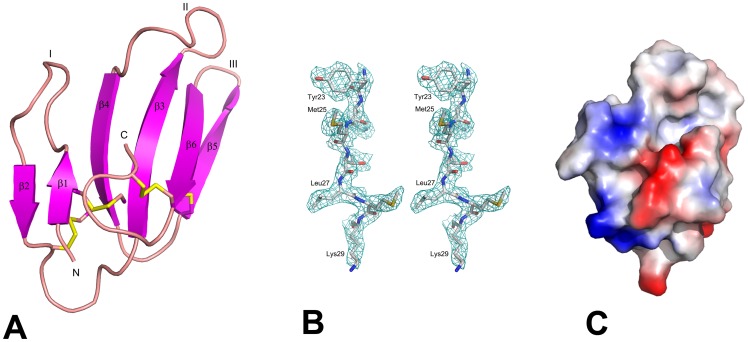
Structure of hemachatoxin. (**A**) Ribbon representation of the hemachatoxin monomer. Cysteine bonds are shown in *yellow.* β-strands, N- and C- terminals are labeled. (**B**) Electron density map**.** A sample final *2Fo-Fc* map of hemachatoxin shows the region from Tyr23 to Lys29. The map is contoured at a level of 1σ. (**C**) The electrostatic surface potential of hemachatoxin is shown in the same orientation as Figure 3A. Blue indicates positive potential and red indicates negative potential in units kT/e. All the structure related figures of this paper were prepared using the program PyMol [77].

**Figure 4:**
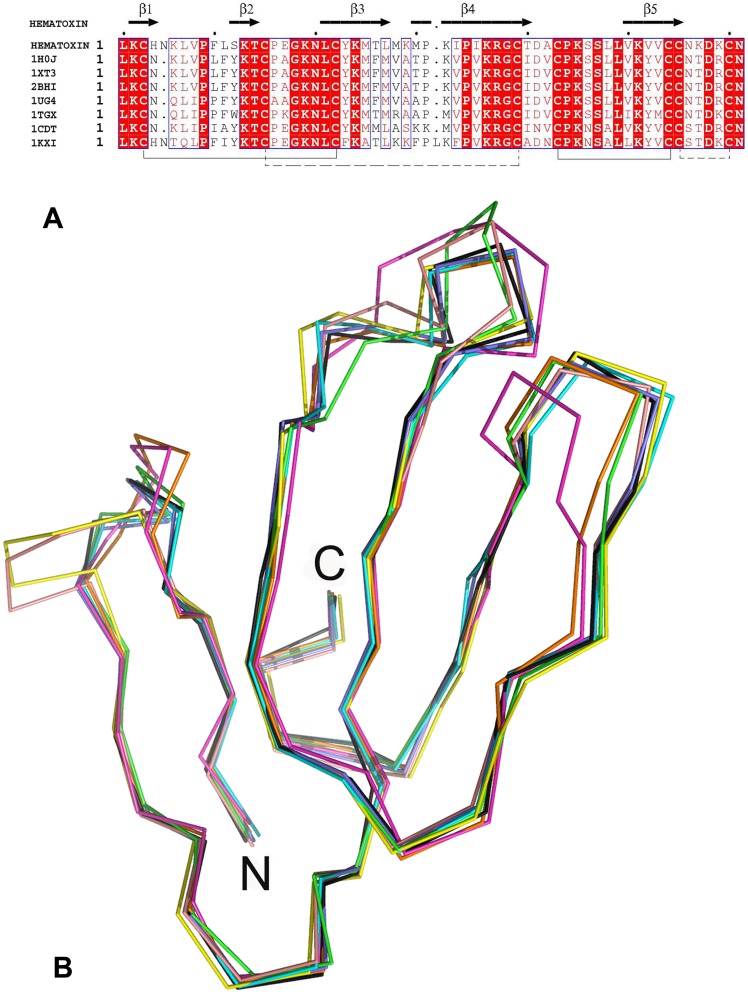
Comparison of hemachatoxin with other three-finger toxins. (**A**) Structure based sequence alignment of hemachatoxin and its homologs, cardiotoxin 3 (1H0J), cytotoxin 3 (1XT3), cardiotoxin A3 (2BHI), cardiotoxin VI (1UG4) and cardiotoxin V (1KXI), (all from *Naja atra*), cardiotoxin V_II_4 (1CDT) from *Naja mossambica* and toxin-γ (1TGX) (a cardiotoxin from *Naja nigricollis*). This figure was generated using the programs ClustalW [78] and ESPript [79]. (**B**) Comparison of hemachatoxin with its structural homologs. Hemachatoxin (brown), cardiotoxin 3 [1H0J] (cyan), cytotoxin 3 [1XT3] (black), carditotoxin A3 [2BHI] (blue), cardiotoxin VI [1UG4] (red), cardiototoxin V [1KXI] (pink), cardiotoxin V_II_4 [1CDT] (green) and toxin-γ [1TGX] (yellow).

**Table 2:** Structural similarity of hemachatoxin with 3FTxs.

Protein	Source	PDB	RMSD*	Z score	Reference
Cardiotoxin V	*Naja atra*	1KXI	1.1 Å(60)	12.2	[49]
Cardiotoxin A3	*Naja atra*	2BHI	0.8 Å(59)	12.0	[50]
Cardiotoxin 3	*Naja atra*	1H0J	0.9 Å(59)	11.7	[51]
Cytotoxin 3	*Naja atra*	1XT3	0.8 Å(59)	11.6	[80]
Toxin-γ	*Naja atra*	1TGX	1.6 Å(59)	11.1	[52]
Cardiotoxin VI	*Naja atra*	1UG4	1.8 Å(59)	11	[81]
Cardiotoxin V_II_4	*Naja atra*	1CDT	1.1 Å(58)	10.5	[82]
Cytotoxin 2	*Naja naja oxiana*	1CCQ	2.1 Å(59)	9.8	[83]
Muscarinic M1 toxin	*Dendroaspis angusticeps*	2VLW	2.4 Å(55)	9.1	[84]
Haditioxin	*Ophiophagus hannah*	3HH7	2.4 Å(58)	8.5	[15]
α-bungarotoxin	*Bungarus multicinctus*	2QC1	2.4 Å(58)	8.4	[85]
Erabutoxin A	*Laticauda semifasciata*	3ERA	2.3 Å(56)	7.9	[86]
Fasciculin 2	*Dendroaspis angusticeps*	1FSC	2.3 Å(55)	7.5	[87]
Toxin FS2	*Dendroaspis polylepis polylepis*	1TFS	2.9 Å(56)	7.4	[65]
Dendroaspin	*Dendroaspis jamesoni kaimosae*	1DRS	3.5 Å(49)	3.6	[66]

* Number of Cα atoms superimposed given in the parenthesis.

## Results

### Isolation and Purification of Hemachatoxin

The *H. haemachatus* crude venom was fractionated on a gel filtration (Superdex 30) column. Peak 3 (Figure 1A) from gel filtration chromatography contained proteins that mostly belong to 3FTx family. Hemachatoxin (*black arrow*) was purified from peak 3 on a C_18_ reverse-phase column (Figure 1B) and further purified to homogeneity using a shallow gradient on the same column (Figure 1C). The homogeneity and mass of hemachatoxin was determined by electrospray ionization mass spectrometry (ESI-MS). ESI-MS showed 3 peaks of mass/charge (*m/z*) ratio ranging from +4 to +6 charges (Figure 1D). The mass was calculated to be 6835.68±0.94 Da.

### Sequence Determination and Analysis

We determined the complete amino acid sequence of hemachatoxin by automated Edman degradation. The first 45 amino acid residues were determined by sequencing the native protein while the remaining sequence was determined from overlapping fragments of chemically-cleaved S-pyridylethylated hemachatoxin (Figure S1A,S1B, S2) (Table S1). The calculated mass of 6836.4 Da from the hemachatoxin sequence agrees well with the experimentally determined molecular mass (6835.68±0.94 Da). The crystal structure (see below) with well defined electron density for the entire hemachatoxin molecule was used to confirm the experimentally determined sequence of the protein as described earlier [41]. The BLAST search [42] showed that hemachatoxin is closely related (>70% identity) to cardiotoxins/cytotoxins, a subgroup of 3FTxs (Figure 2A). Hemachatoxin exhibited highest identity to cytotoxin 1 (97%) [43], cytotoxin 2 (89%) and cytotoxin 3 (84%) [44], purified from *Hemachatus haemachatus* venom. Hemachatoxin differs from cytotoxin 1 [43] in two amino acid positions (Leu27Met28 is replaced by Met27Leu28). This difference was confirmed by ESI-MS (CNBr cleavage site and mass of peptides, Table S1), Edman degradation (Figure S3A, S3B and S3C) and electron density map (see below). Thus hemachatoxin belongs to the 3FTx family based on sequence similarity and the position of cysteine residues (Figure 2).

### Structural Analysis

The structure of hemachatoxin was determined by the molecular replacement method using *Naja nigricollis* toxin-γ coordinates (PDB code 1TGX) as a search model. There were two hemachatoxin molecules in an asymmetric unit with each molecule consisting of residues from Leu1 to Asn61 (Figure 3A). Both monomers are well defined in the electron density map (Figure 3B). The model was refined to a final R value of 0.23 (R_free_ = 0.28) (Table 1). The stereochemical parameters of the model were analyzed by PROCHECK [45] and all residues are in the allowed regions of the Ramachandran plot. Each monomer of the asymmetric unit consists of 6 anti-parallel β-strands (β2↓β1↑β4↓β3↑β6↓β5↑) that form two β-sheets (Figure 3A). The first β-sheet consists of two anti-parallel β-strands, β1 (Lys2-Lys6) and β2 (Phe10-Thr14), while the second contains four anti-parallel strands, β3 (Leu21-Thr26), β4 (Ile35-Thr40), β5 (Ala42-Ser47) and β6 (Lys51-Asn56). The fold of hemachatoxin is maintained by four disulfide bonds, and these cysteines are strictly conserved among the 3FTxs. The three fingers of hemachatoxin consist of the secondary structures β1Ωβ2, β3Ωβ4 and β5Ωβ6 (Figure 3A). The electrostatic surface representation shows that loops I and II are predominantly charged residues, whereas loop III is highly hydrophobic in nature (Figure 3C). The sequence alignment revealed the conserved residues of hemachatoxin as well as its identity to cardiotoxins/cytotoxins (Figure 4A). Also, hemachatoxin shared the common three-finger fold and molecular shape when compared to its structural homologues (Figure 4B) [46].

## Discussion

The three-dimensional structures of snake venom 3FTxs, particularly that of neurotoxins [15], [20], [47], [48] and cardiotoxins/cytotoxins [49]–[52] have been extensively studied. Here we report the structural characterization of a new 3FTx, hemachatoxin from the venom of *H. haemachatus*. The structural analyses indicate that hemachatoxin belongs to cardiotoxin/cytotoxin subgroup of 3FTx family. It exhibited 97% sequence identity to cytotoxin 1 [43], whose crystal structure has not been determined. ESI-MS, Edman degradation and crystal structure data indicates that hemachatoxin differs from cytotoxin 1 in two amino acid positions (Leu27Met28 is replaced by Met27Leu28) and hence are isoforms. Multiple isoforms of 3FTxs are known to be present in single snake venom [53], [54].

As mentioned in the introduction section, 3FTxs, including hemachatoxin, share overall structural similarity (Figure 4B), but they differ from each other in their biological activities. Subtle variations in the size and conformation of β-sheet loops dictate the biological specificities in 3FTxs. For example, the well characterized long-chain (e.g. α-cobratoxin, α-bungarotoxin) and short-chain (e.g. erabutoxin a, toxin-α) neurotoxins that differ in loop size and length of C-terminal extension, exhibit distinct specificity for nAChR subtypes. Short-chain neurotoxins has a longer loop I (12–13 amino acid residues (aa) vs. 9–12 aa in long-chain neurotoxins), a shorter loop II (15–16 aa vs. 19–20 aa in long-chain neurotoxins) and C-terminal tail (2 aa vs. 7–24 aa in long-chain neurotoxins) when compared to long-chain neurotoxins. This longer loop I of short-chain neurotoxins contains key functional residues that are important for recognizing the nicotinic acetylcholine receptor [55], [56], while shorter loop I of long-chain neurotoxins lacks these functional residues. The long C-terminal tail appears to ‘substitute’ for the loop I functional residues and contribute to the receptor binding [57], [58]. The deletion of this C-terminal tail reduces the binding affinity [59], [60]. Similarly, the difference in the conformations of the three loops appears to dictate the biological specificities of these neurotoxins. Both short-chain and long-chain neurotoxins exhibit equi-potency towards muscle αβγδ nAChR [56], [60] but only long-chain neurotoxins, not short-chain neurotoxins, bind to neuronal α7 nAChR with high affinity [61], [62]. Detailed structure-function studies indicate that the presence of the fifth disulfide bond in loop II enables long-chain neurotoxins to recognize α7 nAChR. The short helical segment formed by the fifth disulfide is thought to be crucial for the target receptor recognition [62], [63]. Thus, size and conformation of the loops indeed affects the interaction of neurotoxins with their receptor. Similarly, structures of loop I in fasciculin [64], and loop III in FS2 [65] and dendroaspin [66] have distinct conformations. Hence, subtle conformational differences in the loops of 3FTxs may help in identifying putative functions.

Hemachatoxin shows highest similarity to P-type cardiotoxins [67] (Figure 2A). Similar to these P-type cardiotoxins, hemachatoxin has the conserved Pro31 and cytolytic site. The three-dimensional structure is similar to P-type cardiotoxins (Figure 4B) (RMSD values, 0.8 to 2.1 Å for 58 to 60 Cα atoms; Z score values, 12.2 to 9.8). Besides, hemachatoxin shows considerable structural identity with S-type cardiotoxins (RMSD 1.1 to 2.8 for 58 to 59 Cα atoms; Z score values, 10.5 to 6.3) (data not shown). However, the similarity with other groups of 3FTxs, such as neurotoxins, muscarinic toxins, fasciculin, FS2 or dendroaspin, is relatively low (Figure 2B, Table 2). The P-type cardiotoxins bind to phospholipids and perturb the membrane surface with their lipid binding sites (6–13, 24–37 and 46–50 amino acid positions in the tip of loop I, II and III, respectively) [67]–[69]. These hydrophobic residues flanked by cationic residues form cytolytic region in cardiotoxins [70], [71]. We compared the B values of the cardiotoxin loops with those of hemachatoxin. All three loops in P-type cardiotoxins showed a high B value (an increase of 5–8 Å^2^) compared with the rest of the molecule. A similar increase in B values (upto 8 Å^2^ increase) was also observed in hemachatoxin loops. Only loop II has the crystal contact suggesting that the observed increase in B values might be limited by the symmetry contacts. Nonetheless this analysis suggests that loop II is flexible and may remain flexible until its interaction with membrane phospholipids. These structural analyses also suggest that hemachatoxin might be having cardiotoxic/cytotoxic activity and our future experiments will be directed to characterize the activity of hemachatoxin.

### Conclusion

In summary we report the isolation, purification and structural characterization of a new 3FTx, hemachatoxin from *H. haemachatus* venom. The structural and sequence analysis reveals hemachatoxin to be a P-type cardiotoxin. Close comparison of the loops of hemachatoxin with other 3FTxs suggests that hemachatoxin has structural features similar to the well characterized cardiotoxins. The structural analysis combined with literature predicts hemachatoxin to have cardiotoxic/cytotoxic properties. Additional experiments are required to fully characterize the activity of hemachatoxin.

## Materials and Methods

### Protein Purification

Lyophilized *H. haemachatus* crude venom was purchased from South African Venom Suppliers (Louis Trichardt, South Africa). Size-fractionation of the crude venom (100 mg in 1 ml of distilled water) was carried out on a Superdex 30 gel-filtration column (1.6×60 cm) pre-equilibrated with 50 mM Tris-HCl buffer (pH 7.4). The proteins were eluted with the same buffer using an ÄKTA purifier system (GE Healthcare, Uppsala, Sweden). Peak 3 from the gel-filtration chromatography was sub-fractionated by reverse phase–high performance liquid chromatography (RP-HPLC) on a Jupiter C_18_ column (10×250 mm) equilibrated with solvent A (0.1% TFA). The bound proteins were eluted using a linear gradient of 28–50% solvent B (80% acetonitrile in 0.1% TFA). The mass of each fraction were analyzed on a LCQ Fleet™ Ion Trap LC/MS system (Thermo Scientific, San Jose, USA). Xcalibur™ 2.1 and ProMass deconvolution 2.8 software were used, respectively, to analyze and deconvolute the raw mass data. The peak corresponding to hemachatoxin was pooled and re-chromatographed using a shallow gradient of 35–45% solvent B on the same column. The mass and homogeneity of purified hemachatoxin was analyzed as described above.

### Sequencing

Hemachatoxin (1.2 mg) was dissolved in 500 µl of denaturation buffer (130 mM Tris-HCl pH 8.5, 1 mM EDTA, 6 M guanidine HCl). After the addition of the reducing agent β-mercaptoethanol (1.23 µl; 25×molar excess of disulfide bonds), the reaction mixture was incubated under a nitrogen stream for 3 h at room temperature. Subsequently, the alkylating reagent 4-vinylpyridine (5.7 µl; 3×molar excess of β-mercaptoethanol) was added and incubated under a nitrogen stream for another 2 h at room temperature. The S-pyridylethylated protein was immediately separated from the reaction mixture by RP-HPLC on a Jupiter C_18_ column (4.6×250 mm) using a linear gradient of 20–60% solvent B and the mass was determined by ESI-MS as discussed above. For cyanogen bromide (CNBr) cleavage, the S-pyridylethylated protein (0.82 mg) was dissolved in 410 µl of 70% TFA to which CNBr (67.7 µl in 70% TFA) was added in order to yield a final protein concentration of 1 µg/µl. CNBr was used at a molar ratio to methionine residue of 200∶1. The reaction tube was incubated in complete darkness for 24 h at room temperature. After 24 h, 8.2 ml of Milli-Q water (10× of reaction mixture) was added into the reaction tube and, subsequently, the reaction tube was lyophilized overnight [72]. The lyophilized sample was re-solubilized in 3 ml of 0.1% TFA for separation by reverse-phase chromatography on a Jupiter C_18_ column (4.6×250 mm) using a linear gradient of 10–50% solvent B. The masses of the peptide fragments were determined by ESI-MS (data not shown). The N-terminal sequence of native hemachatoxin and peptides generated by CNBr cleavage (identified by mass spectrometry data) were determined by automated Edman degradation using a PerkinElmer Life Sciences Model 494 pulsed liquid-phase sequencer (Procise, Foster City, USA) with an on-line Model 785A phenylthiohydantoin-derivative analyzer. The complete amino acid sequence of hemachatoxin was determined by overlapping sequences.

### Crystallization and Structure Determination

Crystallization screens were performed with the hanging drop vapor diffusion method using Hampton Research and Jena Bioscience screens. The protein was at a concentration of 35 mg/ml, and 1∶1 crystallization drops were set up with the reservoir solution. The diffraction quality crystals of hemachatoxin were obtained from a reservoir solution containing 150 mM ammonium acetate, 100 mM sodium acetate (pH 4.6) and 25% polyethylene glycol 4000. Crystals were grown up to 10 days and were cryo-protected with 20% (w/v) glycerol supplemented (the mother liquor concentration was maintained by exchanging water with glycerol) with the crystallization condition. Hemachatoxin crystal diffracted up to 2.43 Å resolution and belongs to P2_1_2_1_2_1_ space group. A complete data set was collected using an R-Axis IV^++^ image plate mounted on a rotating anode Rigaku X-ray generator. The data set was processed and scaled using HKL2000 [73]. The structure of hemachatoxin was determined by the molecular replacement method using the program Phaser [74]. The coordinates of *Naja nigricollis* toxin-γ monomer structure (PDB code 1TGX; sequence identity 67%) were used as a search model. The structure solution was obtained with LLG- 94; and TFZ score of 12.3 and RFZ score 4.5. Initial rigid body refinement gave R_work_ 36.6 (R_free_ 43.5). There were two hemachatoxin molecules located in the asymmetric unit. The resultant electron density map was of good quality. Several cycles of model building/refitting using the program Coot [75], and alternated with refinement using the program Phenix [76], lead to the convergence of R-values (Table 1). Non-crystallographic symmetry (NCS) restraints were used throughout the refinement process.

### Accession Numbers

The protein sequence data reported in this paper will appear in the UniProt Knowledgebase under the accession number B3EWH9. The three dimensional coordinates and structure factors of hemachatoxin were deposited in the RCSB (www.pdb.org) database with the access code 3VTS.

## Supporting Information

Figure S1 **Reduction and pyridylethylation of hemachatoxin.** **(A)** The S**-**pyridylethylated hemachatoxin (*black arrow*) was purified on a linear gradient of 20–60% solvent B. **(B)** The ESI-MS profile of S-pyridylethylated hemachatoxin showing the four peaks of mass/charge (m/z) ratio ranging from +4 to +7 charges. The mass was determined to be 7685.12±1.14 Da.
(TIF)

Figure S2 **Separation of peptides derived from cyanogen bromide cleavage of the S-pyridylethylated hemachatoxin on RP-HPLC.** A linear gradient of 10–50% solvent B was used. The peptides A and B were sequenced by Edman degradation method.
(TIF)

Figure S3 **Chromatographic profiles of PTH-amino acid (phenylthiohydantoin-amino acid) residues 27 and 28 of the Edman degradation cycles 29 and 30. (A)** Elution profile of standard PTH-amino acid residues**. (B)** Cycle 29 of Edman degradation showing the 27^th^ residue, PTH-L. PTH-T and PTH-M denotes the carryover from 28^th^ and 27^th^ cycle, respectively. **(C)** Cycle 30 of Edman degradation showing the 28^th^ residue, PTH-M. PTH-L denote the carryover from 29^th^ cycle.
(TIF)

Table S1 **The sequence determination of hemachatoxin.**
(DOC)
