# Supplementary figures and images for: Identification and Structural Characterization of a New Three-Finger Toxin Hemachatoxin from Hemachatus haemachatus Venom
Source: PLoS One. 2012 Oct 29;7(10):e48112. doi: 10.1371/journal.pone.0048112 (PMC3483290; doi:10.1371/journal.pone.0048112)

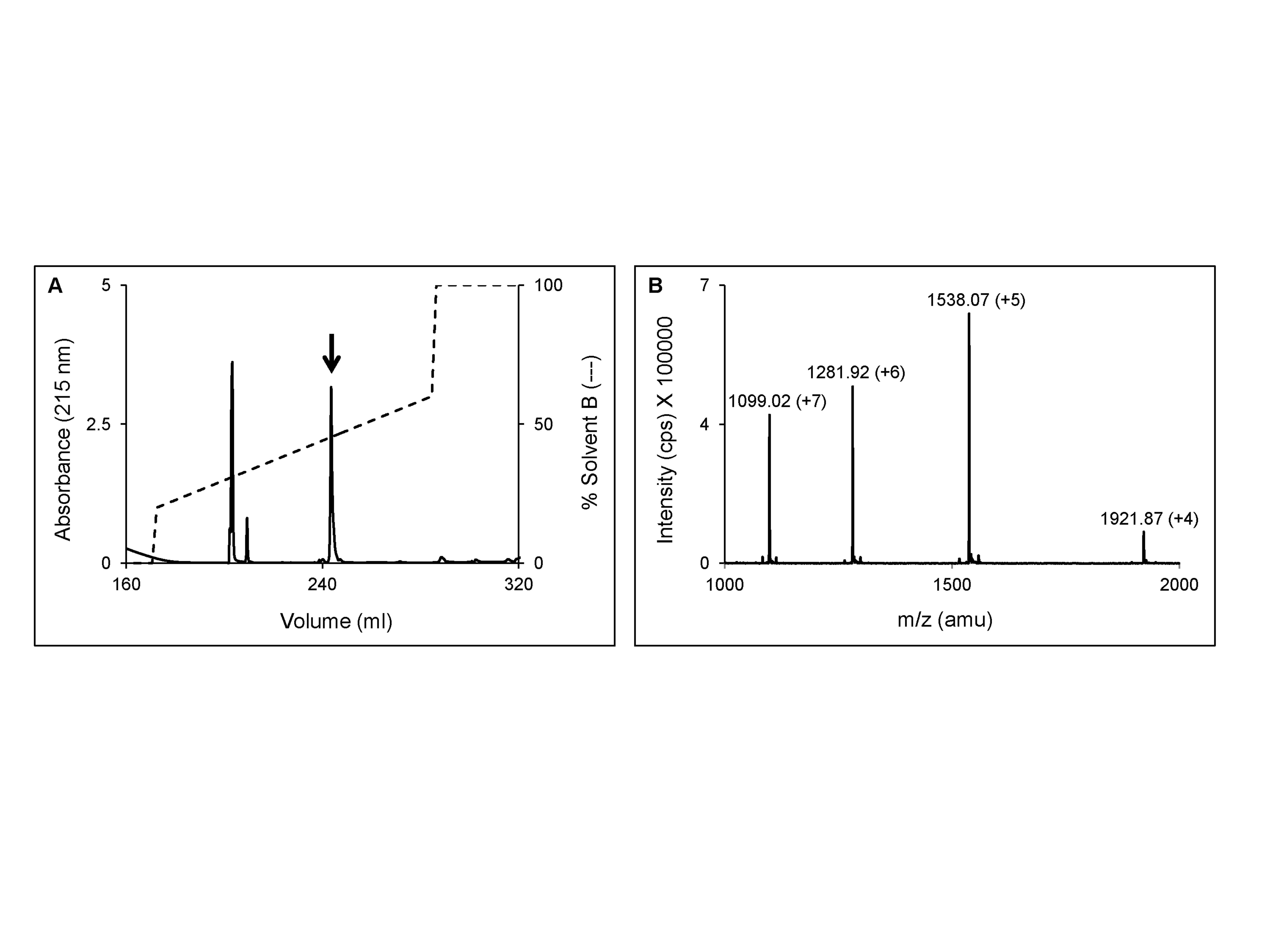

Supplement: Figure S1 — Reduction and pyridylethylation of hemachatoxin. (A) The S-pyridylethylated hemachatoxin (black arrow) was purified on a linear gradient of 20–60% solvent B. (B) The ESI-MS profile of S-pyridylethylated hemachatoxin showing the four peaks of mass/charge (m/z) ratio ranging from +4 to +7 charges. The mass was determined to be 7685.12±1.14 Da. (TIF) [file pone.0048112.s001.tif]

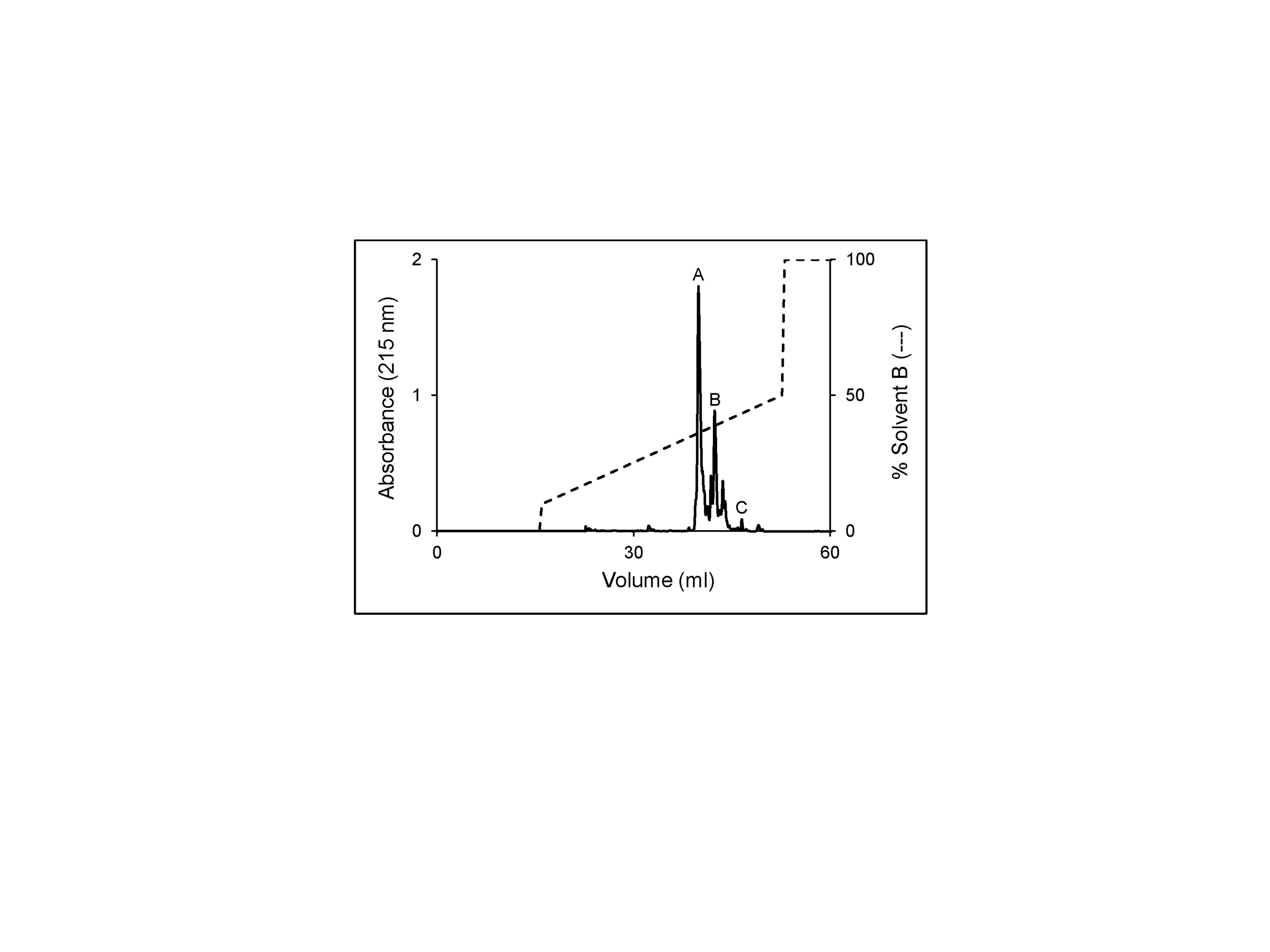

Supplement: Figure S2 — Separation of peptides derived from cyanogen bromide cleavage of the S-pyridylethylated hemachatoxin on RP-HPLC. A linear gradient of 10–50% solvent B was used. The peptides A and B were sequenced by Edman degradation method. (TIF) [file pone.0048112.s002.tif]

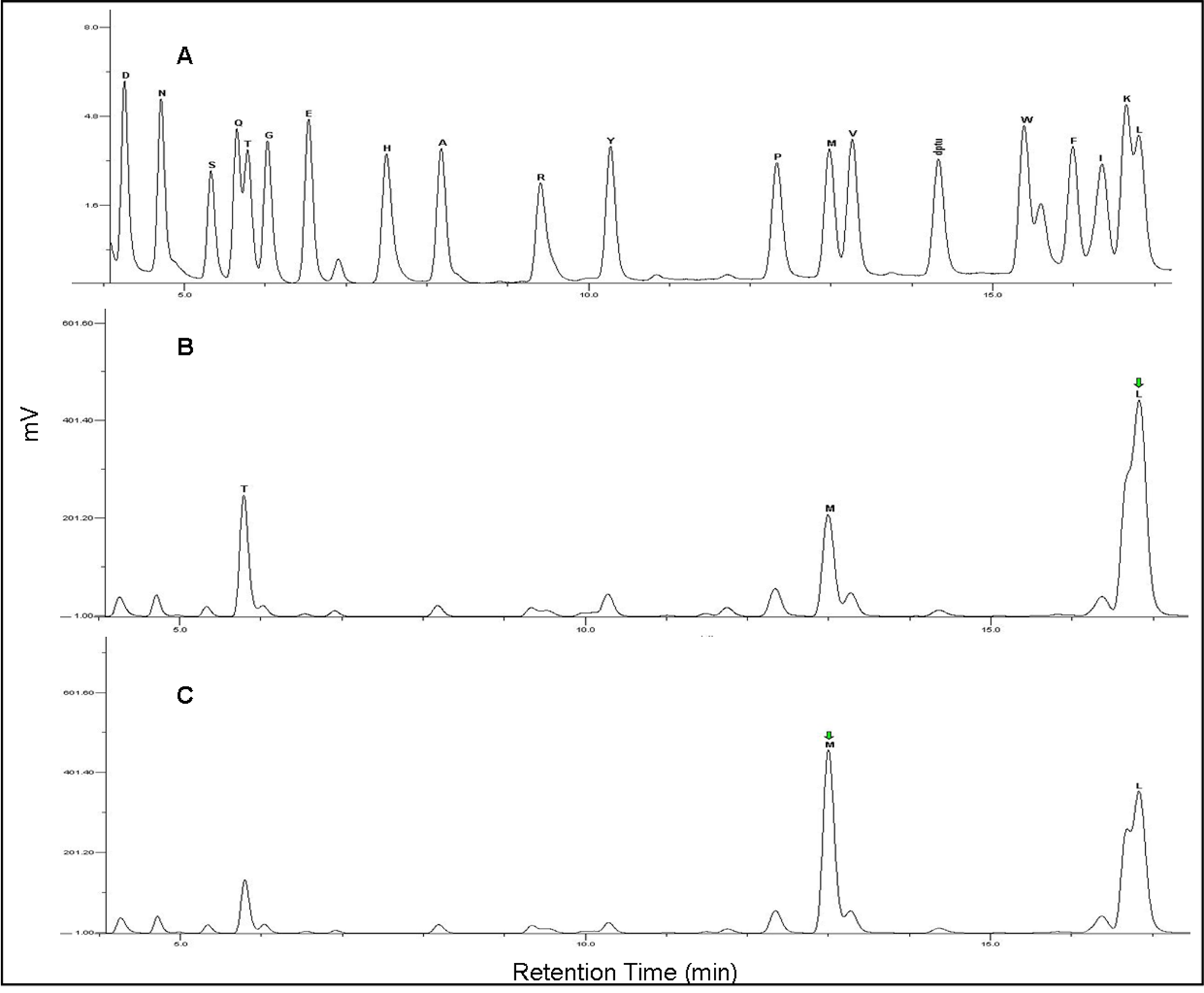

Supplement: Figure S3 — Chromatographic profiles of PTH-amino acid (phenylthiohydantoin-amino acid) residues 27 and 28 of the Edman degradation cycles 29 and 30. (A) Elution profile of standard PTH-amino acid residues. (B) Cycle 29 of Edman degradation showing the 27th residue, PTH-L. PTH-T and PTH-M denotes the carryover from 28th and 27th cycle, respectively. (C) Cycle 30 of Edman degradation showing the 28th residue, PTH-M. PTH-L denote the carryover from 29th cycle. (TIF) [file pone.0048112.s003.tif]
