# Supplementary material for: Identification and Structural Characterization of a New Three-Finger Toxin Hemachatoxin from Hemachatus haemachatus Venom
Source: PLoS One. 2012 Oct 29;7(10):e48112. doi: 10.1371/journal.pone.0048112 (PMC3483290; doi:10.1371/journal.pone.0048112)
Supplement: Table S1 — The sequence determination of hemachatoxin. (DOC) [file pone.0048112.s004.doc]

**Table S1.** The sequence determination of hemachatoxin

| Complete Sequence  Native  Peptide A (31-61)^a^  Peptide B (1-25)^b^  Peptide C (1-28)^#^ | LKCHNKLVPFLSKTCPEGKNLCYKMTLMKMPKIPIKRGCTDACPKSSLLVKVVCCNKDKCN  LKXHNKLVPFLSKTXPEGKNLXYKMTLMKMPKIPIKRGXTDAXPK  PKIPIKRGCTDACPKSSLLVKVVCCNKDKCN  LKCHNKLVPFLSKTCPEGKNLCYKM^*^  LKCHNKLVPFLSKTCPEGKNLCYKMTLM^*^ |
| --- | --- |

^#^based on the estimated mass of peptide C from ESI-MS (3507.8 ± 0.54 Da) which matches with the calculated mass

(3506.83 Da).

^*^denotes the homoserine lactone (as the C-terminal Met is changed to homoserine lactone after the CNBr cleavage).

^a^denotes the mass of peptide A: 3887.9 ± 0.13 Da

^b^denotes the mass of peptide B: 3162.8 ± 0.33 Da
